# Microbial model communities: To understand complexity, harness the power of simplicity

**DOI:** 10.1016/j.csbj.2020.11.043

**Published:** 2020-12-02

**Authors:** Johan Bengtsson-Palme

**Affiliations:** a Department of Infectious Diseases, Institute of Biomedicine, The Sahlgrenska Academy, University of Gothenburg, Guldhedsgatan 10, SE-413 46 Gothenburg, Sweden; b Centre for Antibiotic Resistance Research (CARe) at University of Gothenburg, Gothenburg, Sweden

**Keywords:** Community-intrinsic properties, Interactions, Microbial communities, Model systems, Standardization

## Abstract

Natural microbial communities are complex ecosystems with myriads of interactions. To deal with this complexity, we can apply lessons learned from the study of model organisms and try to find simpler systems that can shed light on the same questions. Here, microbial model communities are essential, as they can allow us to learn about the metabolic interactions, genetic mechanisms and ecological principles governing and structuring communities. A variety of microbial model communities of varying complexity have already been developed, representing different purposes, environments and phenomena. However, choosing a suitable model community for one’s research question is no easy task. This review aims to be a guide in the selection process, which can help the researcher to select a sufficiently well-studied model community that also fulfills other relevant criteria. For example, a good model community should consist of species that are easy to grow, have been evaluated for community behaviors, provide simple readouts and – in some cases – be of relevance for natural ecosystems. Finally, there is a need to standardize growth conditions for microbial model communities and agree on definitions of community-specific phenomena and frameworks for community interactions. Such developments would be the key to harnessing the power of simplicity to start disentangling complex community interactions.

## Graphical abstract

**Figure:**
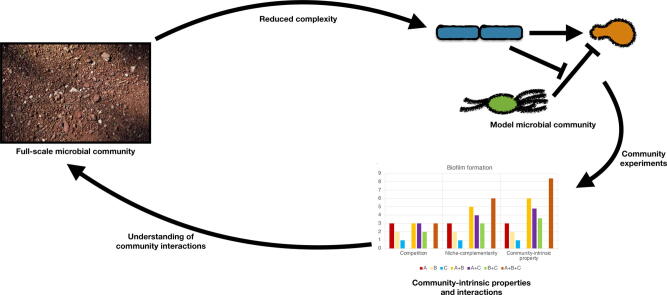

## 1 Introduction

Natural microbial ecosystems are complex. Often, they contain hundreds or thousands of species that show a myriad of interactions, both within and between species, as well as with the surrounding environment. Many of the ecosystem functions in these communities, and by consequence the ecosystem services we exploit, are inherently dependent on such complex interaction networks. One goal of synthetic microbial ecology is to tame these interactions and utilize them for, e.g., biotechnological or medical purposes [1], [2], [3], [4], [5], [6]. In order to do so, it is imperative to maintain the key microbial populations that keep these beneficial processes active, as well as the microbial species and environmental factors that support growth and stability of these key populations. However, our current understanding of these interactions – or even the governing principles behind microbial interactions in general – is severely limited, in large part simply because microbial interactions are often complex and complicated to study.

One of the most powerful tools in the scientific arsenal is the use of simpler and more accessible models that serve as representatives of more complicated systems. This has been the reasoning behind our use of a wide range of model systems, including *Escherichia coli* as a model for bacterial metabolism and molecular biology more generally [7], *Saccharomyces cerevisiae* as a model for the eukaryotic cell [8], *Drosophila melanogaster* as a model for embryonic development [9], and *Caenorhabditis elegans* as a model of neuronal development [10]. The remarkable extent to which we have been able to learn and extrapolate from these simplistic model systems conveys fundamental information regarding how the biological world is organized. The evolutionary processes that have led to the diversity of life we observe today offer plenty of symmetries and similarities that we can exploit to better understand all living organisms.

There are several benefits of using models rather than the system primarily of interest. First, model systems provide settings where far more parameters can be controlled and maintained over time, and where replication of experiments is made much easier. Second, model systems are generally less complex; an ideal model system should be sufficiently complex to answer the question at hand, but not more complicated than required. Third, most model systems offer increased ease of experiments, such as shorter generation times, simpler genetics, less demanding growth conditions, and lower costs. With these factors in mind, the same principles of reduction can be applied to microbial communities as well, in order to understand the interactions driving ecosystem processes and manipulate their outcomes. In this paper, the various existing microbial model communities will be compared and their feasibility in terms of ease of use, readouts and ability to capture community-intrinsic properties and interactions will be discussed. The paper will not discuss computational and mathematical modeling of microbial communities in detail, as this has been excellently done recently by others [11], [12], [13].

## 2 What constitutes a good microbial community model?

Many microbes can be reproducibly grown in the lab under precisely controllable conditions. In addition, they are single-celled and have fairly well-defined metabolism and lifecycles but can at the same time show complex interaction behaviors. On top of that, a long range of bacteria are easy to quickly grow in the lab at large-scales and are amendable to genetic manipulation. These aspects together position communities of microbes as seemingly perfect models to study ecological and evolutionary principles as well as the genetic mechanisms governing interspecies interactions [14]. Furthermore, understanding of microbial communities has direct applications in biotechnology, health and agriculture [2], [3], [5], [15], meaning that these microbial models may provide both theoretical knowledge and direct practical applications of biology.

Still, there has been considerable debate regarding the value of microbial model communities in terms of generating actual biologically relevant knowledge [16]. The experimental systems have been (rightly) accused of being highly artificial, the relevance of synthetic communities or reduced-diversity selection of organisms from a natural community has been questioned, the dramatically smaller number of possible interactions in a four-species community compared to one with thousands of species may result in important connections being lost, and so forth. There are also the questions of to what extent microbial ecology follows the same principles as the ecology of macroorganisms [17], [18] and if model systems capture the aspect of scale in ecology [19]. While much of this critique is relevant, some of it also misses the greater points; two main purposes of investigating microbial model systems are to deduce if there are general mechanisms in small-scale communities that also apply to real-world complex systems, and to generate mechanistic hypotheses that can be tested in more complex settings. Model communities could, for example, be used to elucidate how microorganisms share metabolites and partition resources [20], [21], [22], under what conditions communities are dominated by competitive or cooperative interactions [23], [24], [25], which specific genes that govern community interactions through e.g. secondary metabolites [26], [27], and to what extent interactions are dependent on environmental changes [28]. Furthermore, model systems can aid in understanding the links between taxonomic and functional diversity [29], what factors that contributes to community stability in the face of invasion from non-resident strains or other stressors [30], [31], highlight important keystone species for functional stability of a community [32], [33], and shed light on the evolution of mutualism between microbes [34], [35]. In addition, microbial model communities can address specific concerns in human health (what allows a pathogenic strain to outcompete less harmful bacteria) [36], [37] or what a minimal set of species would be to maximize production of an industrially important compound [38], [39]. The simplistic nature of these microbial model communities allows testing of hypotheses under controlled conditions, while also permitting manipulation of the organisms’ genomes, enabling mechanistic understanding of ecological patterns and interactions [40]. This mechanistic understanding can then be used when interpreting the results of large-scale analyses of real-world systems, using e.g. metagenomic, transcriptomic or proteomic data [41], [42]. Pessotti et al. have suggested criteria for model ecological systems that are very worthwhile to consider in this context: (i) they should have an easily detectable output that indicates healthy microbiome function, (ii) they should contain microbial members that are culturable and genetically tractable, and (iii) the community behaviors should be readily recapitulated in a laboratory setting and scalable to higher throughput [40]. The authors also list two criteria relating to being based on a natural system, which has advantages from a direct relevance point of view, but is more debatable as general criteria for selecting a model system, as this would depend on the objectives of the research [43]. In addition, these criteria could be supplemented with other properties that a model community would need to have to answer certain research questions. However, these criteria would generally not be broadly applicable to all uses of microbial model communities. For example, a microbial model community that will be used to assess interactions with the host in the human gut needs to be able to grow anaerobically and probably need to be able to attach to epithelial surfaces. Similarly, if one wants to investigate the effect of species removal on the relation between taxonomic and functional diversity, one needs to select a sufficiently diverse model community to begin with. In the overview of microbial model communities below, the three criteria listed above have been considered in the selection process and the origin of the community has been noted so that the reader can make up their own mind regarding the relevance of natural versus synthetic communities based on their research questions.

It should be noted that while all the models described in this paper contain members that are fairly easily cultivable under laboratory conditions, not all of these microbes are equally easy to genetically manipulate, which is an important consideration when selecting a model system [14], [40]. Furthermore, they are all scalable to some degree and most are easy to adapt to high-throughput experiments at least for some readouts. In one respect these model communities differ from one another; not all of the suggested models in the literature have an output that is easy to measure that indicates whether the community is performing ‘normally’, is stable relative to the expected outcome and whether interactions take place in the community model. Such outputs can be relatively simple, such as degree of biofilm formation, production of certain molecules, growth in the absence of some essential metabolite, or increased tolerance to certain chemicals. Relative abundances of the community members can also be used to detect community stability, but is a less reliable and/or informative indicator of interactions and community health than the former readouts.

## 3 Community models – From simple to complex

**Fig. 1:**
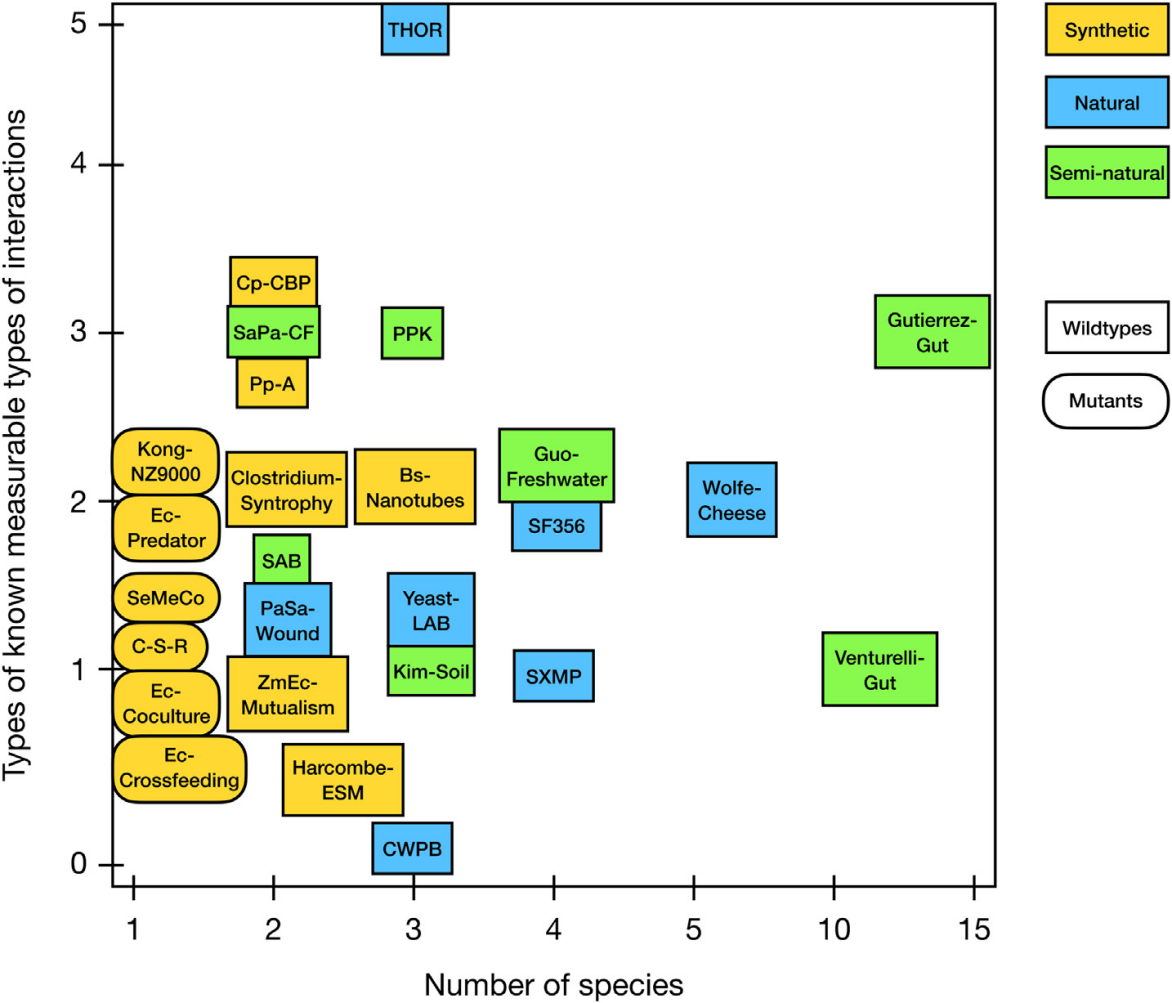
Stratification of microbial model communities by number of species in the model community and number of different types of measurable interaction behaviors as listed in the original publication of the model community (more measurable interactions may have been discovered since the original publication). For details on the communities, see Table 1.

Already, there are a long range of microbial community model systems that have been developed for different purposes (Table 1). Some of these have found uses outside of the original studies they were developed in, while others were designed to answer a more specific research question and have so far found little use outside of that specific setting [44]. Roughly speaking, the available model systems can be divided by a few different criteria: (i) whether it is of natural or synthetic origin [40], [43], (ii) if its members lack the ability to produce essential metabolites that are produced by other members of the community [25], (iii) the number of species in the community, and (iv) the type of known interactions in the community (Fig. 1). This division essentially creates three groups of model communities: the mutant-based communities, the multispecies synthetic communities and the (semi-) natural communities.

**Table 1:** Overview of microbial model communities. Community designations have been taken from the original publications whenever possible; otherwise designations have been created for the communities in order to be able to reference them in the text. This table is a collection of the author’s best effort to accurately collect data from the source papers for each community. This may not be an exhaustive list of all microbial model communities that exist, nor may it be complete in terms of e.g. measurable interactions. That said, the table can serve as a guide to which model communities that may be suitable for certain purposes.

Community designation	Authors	Year	No. species	Species	Basis	Combination strategy	Known interactions	Measurable behaviours	
SXMP	Ren et al.	2014	4	*Stenotrophomonas rhizophila, Xanthomonas retroflexus, Microbacterium oxydans, Paenibacillus amylolyticus*	Agricultural bacterial isolates	Best biofilm-forming capacity among combinations of 7 different agricultural bacterial isolates	Increased biofilm formation	Biofilm formation	[45]
PPK	Lee et al.	2014	3	*Pseudomonas aeruginosa, Pseudomonas protegens, Klebsiella pneumoniae*	Biofilm isolates	Commonly co-occuring, biofilm-forming bacteria	Potential metabolic cooperation	Resistance to tobramycin and SDS, Biofilm formation	[28]
SF356	Kato et al.	2005	4	*Clostridium straminisolvens CSK1, Clostridium* sp. *strain FG4, Pseudoxanthomonas* sp. *strain M1-3, Brevibacillus* sp. *strain M1-5, Bordetella* sp. *strain M1-6*	Cellulose-degrading defined mixed culture	The five dominant bacterial strains in an enrichment culture capable of degrading cellulosic materials	Metabolic cooperation (described substrate flows)	Paper degradation, accumulation of oligosaccharides, acetate and ethanol (unclear how the community differs to single-strains though)	[38]
Wolfe-Cheese	Wolfe et al.	2014	6	*Staphylococcus*)*, Brevibacterium JB5, Brachybacterium JB7, Candida 135E, Penicillium JBC, Scopulariopsis JB370*	Cheese rinds	Most common members from a large study of cheese rind diversity	pH regulation	Pigment production	[46]
SaPa-CF	Filkins et al.	2015	2	*Staphylococcus aureus, Pseudomonas aeruginosa*	Cystic fibrosis lung co-infections	Described co-culture of model members in cystic fibrosis patients from the literature	Coexistance, competition	*S. aureus* fermentation	[47]
Yeast-LAB	Ponomarova et al.	2017	3	*Saccharomyces cerevisiae, Lactococcus lactis, Lactobacillus plantarum*	Fermented food	Described symbiosis between model members from the literature	Yeast-bacteria metabolic cooperation (amino acids)	Bacterial growth in presence of yeast	[48]
Guo-Freshwater	Guo & Boedicker	2016	4	*Escherichia coli, Aeromonas veronii, Aeromonas hydrophila, Shewanella oneidensis*	Freshwater isolates	Four known freshwater isolates, three of which were from the Los Angeles area	Metabolic cooperation and competition	Metabolic activity	[49]
Gutierrez-Gut	Gutiérrez & Garrido	2019	14	*Escherichia coli, Lactobacillus plantarum, Flavonifrator plautii, Ruminococcus gnavus, Lachnoclostridium symbiosum, Lachnoclostridium clostridioforme, Bifidobacterium adolscentis, Bacteroides dorei, Bacteroides vulgatus, Bacteroides fragilis, Bacteroides cellulosilyticus, Bacteroides ovatus, Bacteroides thetaiotaomicron, Bacteroides finegoldii*	Human gut isolates	Species commonly found in the human gut with sequenced genomes	Metabolic cooperation	Short-chain fatty acid production, relative growth, inulin consumption	[32]
Venturelli-Gut	Venturelli et al.	2018	12	*Bacteroides thetaiotaomicron, Bacteroides ovatus, Bacteroides uniformis, Bacteroides vulgatus, Blautia hydrogenotrophica, Collinsella aerofaciens, Clostridium hiranonis, Desulfovibrio piger, Eggerthella lenta, Eubacterium rectale, Faecalibacterium prausnitzii, Prevotella copri*	Human gut isolates	Species selected to mirror the functional and phylogenetic diversity of the human gut and contribute significantly to human health and disease	Metabolic cooperation and competition	Metabolic activity, relative growth	[22]
Clostridium-Syntrophy	Charubin & Papoutsakis	2019	2	*Clostridium acetobutylicum, Clostridium ljungdahlii*	Industrial applications	Syntrophy between two industrially important *Clostridium* species	Reciprocal syntrophy	2-propanol and 2,3-butanediol production, *C. ljungdahlii* growth in glucose	[50]
Kim-Soil	Kim et al.	2008	3	*Azotobacter vinelandii, Bacillus licheniformis, Paenibacillus curdlanolyticus*	Soil	Designed to survive under nutrient-limited conditions (no evidence for natural interaction)	Reciprocal syntrophy	Population stability in spatially separated conditions	[51]
THOR	Lozano et al.	2019	3	*Pseudomonas koreensis, Flavobacterium johnsoniae, Bacillus cereus*	Soil co-isolates	*Bacillus cereus* “hitchhikers” (co-isolates) showing competitive and synergistic interactions between each other	Increased biofilm formation, inhibition, nutritional enhancement, protection from growth inhibition	Colony expansion, biofilm formation, koreenceine accumulation	[52]
Ec-Predator	Balagaddé et al.	2008	1	*Escherichia coli*	Synthetic	Two genetically modified *E. coli* populations (modified to become predator and prey)	Inhibition, protection from inhibition	Relative growth (induction of inhibition and protection possible)	[23]
SeMeCo	Campbell et al.	2016	1	*Saccharomyces cerevisiae BY4741*	Synthetic	A strain deficient in certain metabolic functions is complemented with plasmids encoding these functions. The plasmids are gradually lost, creating a metabolically diverse population	Metabolic cooperation	Loss of metabolic function	[21]
Pp-A	Christensen et al.	2002	2	*Pseudomonas putida, Acinetobacter strain C6*	Synthetic	Two species able to utilize benzyl alcohol as their sole carbon and energy source	Competition, metabolic cooperation	Biofilm formation, relative growth, physiological activity	[53]
Bs-Nanotubes	Dubey & Ben-Yehuda	2011	2–3	*Bacillus subtilis, Staphylococcus aureus*	Synthetic	Investigation of nanotube formation	Nanotube formation, molecule exchange	Nanotube formation (microscopy)	[54]
Harcombe-ESM	Harcombe et al.	2014	2–3	*Escherichia coli, Salmonella enterica, (Methylobacterium extorquens)*	Synthetic	No known previous interspecies interaction	Metabolic cooperation	Relative growth	[35]
C-S-R	Kerr et al.	2002	1	*Escherichia coli BZB1011*	Synthetic	Three *E. coli* strains (one colicin-producer, one with colicin-resistance mutations and one wildtype), forming a “rock-paper-scissor” relationship	Inhibition, competition	Relative growth	[55]
Kong-NZ9000	Kong et al.	2018	1	*Lactococcus lactis*	Synthetic	Engineered strains of *L. lactis* showing commensalism, amensalism, neutralism, cooperation, competition and predation in pairs	Signaling, antibiotic inhibition	Relative growth (fluorescent markers)	[56]
ZmEc-Mutualism	Kosina et al.	2016	2	*Zymomonas mobilis, Escherichia coli*	Synthetic	Two industrially important species without known ecological interactions	Inhibition, metabolic cooperation	Relative growth	[57]
SAB	Villa et al.	2015	2	*Synechocystis PCC 6803, Escherichia coli K12*	Synthetic	Representatives of the communities formed on limestone stone-air interfaces	Longterm biofilm coexistance	Biofilm formation	[58]
Ec-Coculture	Zhang & Reed	2014	1	*Escherichia coli*	Synthetic	Two *E. coli* auxotrophs grown in co-culture	Metabolic cooperation	Growth in minimal media	[59]
Cp-CBP	Zuroff et al.	2013	2	*Clostridium phytofermentans, Candida molischiana/Saccharomyces cerevisiae*	Synthetic	Species selected for their ability to ferment cellodextrins (consolidated bioprocessing)	Respiratory protection, metabolic cooperation	Ethanol production, cellulose fermentation	[39]
Ec-Crossfeeding	Mee et al.	2014	1	*Escherichia coli*	Synthetic (metabolic crossfeeding)	Engineered *E. coli* mutants with auxotrophic phenotype of 1 of 14 essential amino acids	Metabolic cooperation	Relative growth	[60]
PaSa-Wound	DeLeon et al.	2014	2	*Pseudomonas aeruginosa, Staphylococcus aureus*	Wound infection isolates	Species commonly co-present in wound infections	Antibiotic tolerance	Antibiotic tolerance	[36]
CWPB	Sun et al.	2008	3	*Pseudomonas aeruginosa, Enterococcus faecalis, Staphylococcus aureus*	Wound infection isolates	Three of the most important species associated with multispecies biofilms clinically seen in wound infections	Unclear	Relative growth	[37]

The mutant-based communities are distinguished by being based on a single species, of which several mutants are introduced, usually deficient in some key metabolic function (SeMeCo [21], Ec-Coculture [59], Ec-Crossfeeding [60]) or producing some combination of toxin and tolerance mechanism (Ec-Predator [23], C-S-R [55]). These models are by design not very complex and are generally not intended to reflect real world situations, but rather to answer specific questions related to metabolic interactions and predatory relationships, such as to what degree dispersal influences biodiversity, the stability of predator–prey relationships and how and when a community show cooperative dynamics. Notably, what sets these communities apart from other model systems using mutants to address specific research questions is in essence the focus on ecological interactions and concepts. Indeed, there is a very fine line between the model communities mentioned above and systems to measure, e.g., horizontal gene transfer of plasmids between two different strains, and many such experimental systems occupy a grey-zone adjacent to community research.

The synthetic communities are built from organisms not known to interact or co-inhabit the same environment in nature, which have been combined either in order to force novel interactions to take place, or to exploit potentially beneficial interactions expected from what is known about the individual community members, for example for industrial purposes. The semi-natural and natural communities are a large and heterogenous group of model systems. This group encompasses communities based on various ecosystems, such as soil (THOR [52], Kim-Soil [51], SXMP [45]), water (Guo-Freshwater [49]), cheese rinds (Wolfe-Cheese [46]), the human body (SaPa-CF [47], PaSa-Wound [36]) and industrial settings (SF356 [38]). Lastly, this group also contains two different model systems based on bacteria in the human gut. It is interesting to note that this medically important type of environment is this far only represented by two relatively complex and species rich model communities, despite that e.g. soil is arguably a much more diverse type of environment in terms of microbial species present [61] and is yet only represented by model communities consisting of three to four species..

### 3.1 Mutant-based model communities

Arguably the most simplistic and reductionist type of microbial model communities are those that are based on mutants of a single species. These mutants are often constructed in such a way that they are auxotrophic, i.e. that they are dependent on the metabolic output of the other strains in order to grow. This allows a high degree of control over the experimental system, but at the expense of losing almost any direct relevance to natural microbial communities. That said, for the purpose of studying general interaction phenomena, such as partitioning of metabolic tasks and establishment of dependence structures in communities, the approach is highly suitable, as interaction patterns can be directly connected to clearly defined traits in the form of auxotrophy. A related, but different, approach was taken by Kong et al. [56], who constructed six different pairs of *Lactococcus lactis* strains showing different types of interactions. These pairs were then mixed with strains from the other interaction types to engineer certain outcomes. This type of approach is promising for future ecosystem engineering efforts, although it very specifically is based upon well-established interactions built into the system in advance. It is notable that except for the Kong-NZ9000 communities, these mutant-based systems have almost exclusively been developed using well-known model organisms such as *E. coli* and *S. cerevisiae*, testament to the origins of this approach in classical genetics and molecular biology. Importantly, the mutant-based model communities have a drawback in that the members only differ in pre-defined mutations, which limits the amount of novel interactions that can be discovered using this approach. Furthermore, while these methods can help generate hypotheses regarding between-species interactions, they do not really provide any data towards understanding of multispecies relationships, as the mutant-based communities generally only comprise mutants of a single species.

### 3.2 Multi-species synthetic model communities

Another set of highly artificial communities are those based on assemblages of different species of microbes that have certain properties that make their potential interactions interesting but that are not known to cooccur naturally. This category encompasses communities such as Pp-A [53], consisting of two species that can utilize benzyl alcohol as a carbon source, Bs-Nanotubes [54], in which the members are selected based on their ability to form nanotubes together with *Bacillus subtilis*, Clostridium-Syntrophy [50], which show several potentially valuable industrial properties in co-culture, and Cp-CBP [39], where the members were selected for their ability to ferment cellodextrins. In many of these instances, scientists have tried to force these microbes into community behavior out of a desire to increase biotechnological yields. There are also a few examples of model communities chosen to avoid any known interactions between the members, in order to be able to study the emergence of novel interactions in communities, such as in the cases of Harcombe-ESM [35] and ZmEc-Mutualism [57]. This type of model community is ideal for studying the evolutionary processes leading to mutualisms between different species. A practical benefit of synthetic model communities is that their members can be selected in such a way that they are easy to genetically manipulate and cultivate in the lab, which may not always be the case with model communities based on naturally interacting microbes.

### 3.3 Soil-based model communities

We will next examine the diversity of semi-natural and natural model communities, based on the specific habitat or microbial lifestyle of their source organisms. There are, first of all, model communities aiming to recapture interactions in soil, including SXMP [45] and THOR [52] comprising members known to interact in nature, and Kim-Soil [51] where members are not known to interact naturally. Both the members of SXMP and THOR were partially selected on ability to form biofilms and both also have increased biofilm formation as an easily measured readout of community interactions. That said, they comprise completely non-overlapping sets of members; SXMP consists of *Stenotrophomonas rhizophila*, *Xanthomonas retroflexus*, *Microbacterium oxydans* and *Paenibacillus amylolyticus*, while THOR is built on *Pseudomonas koreensis*, *Flavobacterium johnsoniae* and *Bacillus cereus*. Arguably, the members of THOR would be easier to genetically manipulate using standard protocols, but that has not prevented the SXMP community to be used in a variety of studies [30], [42], [62], [63], [64], [65], compared to THOR which was more recently introduced as a model [26], [52]. The ease of genetic manipulation and the many known interactions between the species in these model communities make them ideal for identifying which genes that are responsible for interaction behaviors between microbial species. However, if any of these two model communities describe interactions in soil more accurately, or if they complement each other, is currently unknown. The Kim-Soil community is interesting in that its members were selected on the criteria of being able to grow under nutrient-limited conditions. The authors of the study then used the community to study interactions via dispersal of molecules between spatially separated populations of the three member species [51].

### 3.4 Biofilm-based model communities

Aside from the soil bacterial model communities forming biofilms described above (SXMP and THOR), there are also model communities that have been selected explicitly on their ability to form biofilms, specifically PPK [28] and SAB [58]. PPK is based on three species that are commonly found together in multi-species biofilms: *Pseudomonas aeruginosa*, *Pseudomonas protegens* and *Klebsiella pneumoniae*. This community is actually one of the most well-studied model communities, although most of the research done on it emanates from the same research constellation [29], [31], [66]. The relative simplicity of the system and the amount of information that can be extracted from how these three microorganisms interact still make this one of the more appealing model systems for microbial interactions. For example, the PPK community has been used to study community tolerance to antimicrobials [29], multi-species spatial organization of biofilms [28] and the role of specific polysaccharides in biofilm community assembly [31]. SAB, on the other hand, is a very specific community model, designed to mimic the relationships between microbes living on the limestone-air interface. It is an interesting model in that it features a cyanobacterium (*Synechocystis* PCC 6803) and a commonly used lab strain of *E. coli* (K12), setting it apart from most other model communities in that it contains a primary producer.

### 3.5 Industrial model communities

Thanks to the great interest in utilizing consortia of microbes to improve biotechnological processes, there are a number of microbial model communities based on or mimicking food production or industrial process applications. These include the cellulose-degrading community SF356 [38], the cheese rind community Wolfe-Cheese [46], the Yeast-LAB [48] community isolated from fermented food, as well as the synthetic communities Pp-A [53] and ZmEc-Mutualism [57] described earlier. SF356 is interesting because it, owing to its industrially useful properties, has quite easily measurable community behaviors: it can degrade paper and accumulates oligosaccharides, acetate and ethanol. On the other hand, none of the members of SF356 is that commonly used for genetic studies, so the ability to manipulate the genomes of these strains is a large uncertainty with this community model. The two food-based communities both incorporate interkingdom interactions between fungi and bacteria. The Yeast-LAB community is selected in such a way that the bacteria are dependent on *S. cerevisiae* to grow, providing a very simple readout for community interactions. The Wolfe-Cheese community is the most species-rich non-human associated model community described here, comprising six different microbial species. A diversity of species allows for more complex interactions, but unless there is a sufficiently good way to disentangle these interactions, this may be more of a problem than a benefit. At the same time, a large number of different species in the model community makes it possible to investigate the impact of removing or replacing certain species on the impact on functional stability. Importantly, a common component of all these industrial and food-based model communities is that any results that come out of studies of their interactions may have direct relevance for biotechnological applications.

### 3.6 Model communities of the human microbiome

**Fig. 2:**
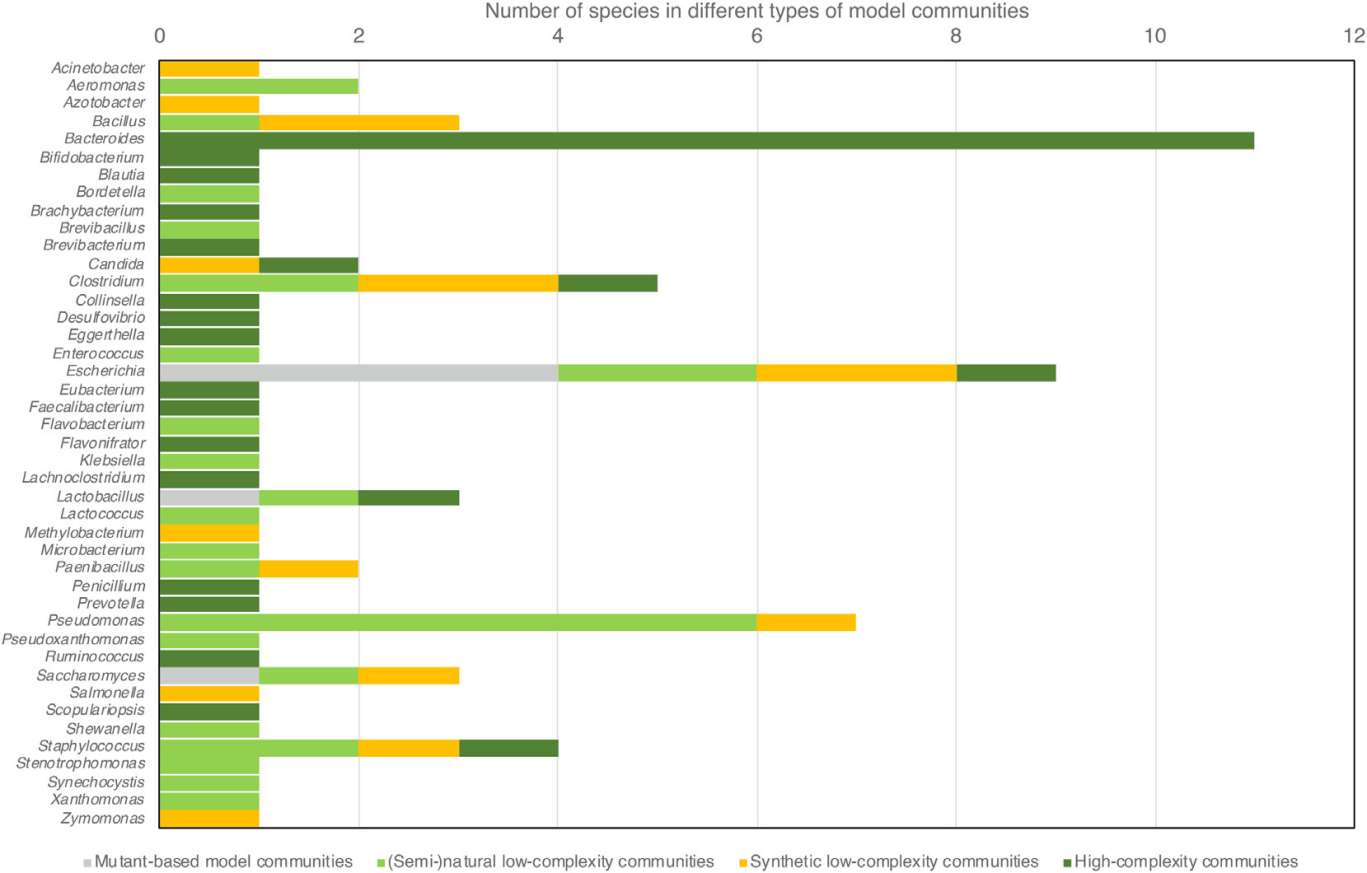
Representation of different genera across the microbial model communities identified in Table 1. Note that each genus can be represented by more than one species in a single model community, such as for *Bacteroides* and *Pseudomonas*, inflating the number for that genera. The purpose of the figure is to show a picture of the taxonomic distribution of current microbial model communities.

One of the major incitements to develop microbial model communities is the desire to understand how interactions between microbes affect human health. It is therefore not surprising that many model communities are based on the human microbiome (Gutierrez-Gut [32], Venturelli-Gut [22]) or human pathogens (SaPa-CF [47], PaSa-Wound [36], CWPB [37]). The two most species-rich model communities described here are based on the human gut microbiome. There are a few important observations related to these two communities. First, despite encompassing many different species – 23 in total – only three are shared between both model communities (*Bacteroides thetaiotaomicron*, *Bacteroides ovatus* and *Bacteroides vulgatus*). This begs the question to what extent these model communities actually mirror the human gut microbiome and whether these 12 to 14 species are needed to capture the most important interactions in the gut, both questions that currently remain unanswered. Second, these two communities are among the more complicated systems to work with, due to the requirement of a low-oxygenic atmosphere for most of the community members to grow. This fact is hard to get around if one aims for any relevance to the human gut ecosystem, but is worth consideration as part of the choice of model system. Third, the overrepresentation of one genus (*Bacteroides*) in these model communities makes them somewhat different on a structural level to many of the other microbial model systems (Fig. 2). It is plausible that the large intra-genus diversity of these model communities may foster more competitive relationships than in other model systems with less species from the same closely related group of bacteria [67], [68]. Such relationships would be interesting to study further, both from a methodological point of view, but also from a perspective of how taxonomic structuring drives competition and cooperation.

The pathogen-based communities all have in common that they contain *P. aeruginosa* and *Staphylococcus aureus*. In addition, the CWPB community also contains *Enterococcus faecalis*. PaSa-Wound and CWPB are both based on wound-infection isolates, while SaPa-CF is based on isolates from cystic fibrosis lung infections. These are all highly relevant infection models, but they may be lacking in terms of beneficial microbes mitigating infections. The fact that they are based around the same, biofilm-forming, species is telling, as it shows the importance of biofilm interactions between pathogenic microbes in causing human disease. One should remember that e.g. the PPK model also comprises human opportunistic pathogenic bacteria, including *P. aeruginosa* [28]. The ability to use community models to predict disease outcomes is one of the key allures of building and studying microbial model communities, but much of this power is yet to be realized.

### 3.7 Other alternative model systems for communities

All of the model systems described this far are well-defined in terms of which species and strains they contain, which – at least in theory – allows clear definition of measurable outputs, specificity in which microbes that are interacting with each other, and the possibility to genetically engineer one or more members of the model community to alter these interactions and outputs. Furthermore, using a defined set of starting strains or mutants enables a great degree of reproducibility, compared to e.g. starting with similar, but not identical, inocula from environmental settings. That said, there are examples of community model systems and methods that are highly useful and interesting for studying community interactions, despite not being as clearly defined. One such method is the *E. coli* mutant system introduced by Wintermute and Silver [20], in which different fluorescently labeled deletion mutant strains are grown together in pairs to identify synergistic interactions. This concept is similar to many of the mutant-based model communities, but is designed to uncover a very specific type of interactions. Another method that could be used to identify community-intrinsic properties (see below) at the community assembly level is the approach used by Goldford et al. [69], in which diverse natural microbial communities are transferred to minimal media, which reduces their diversity in a predictable manner. Potentially, this approach could be used to design model communities, but as it is clear that the choice of medium has a strong sorting effect on which microbes that remain such a design process has to be carefully crafted. Finally, there are also different approaches to measure community responses to stressors, such as antibiotics. These methods generally use some type of environmental inoculum that is similar over time, although not identical, and introduce it to an artificial system allowing for e.g. exposure studies in controlled settings [70], [71]. While these systems in principle allow for detection of interactions between the members, the readouts are fairly complex and it is often hard to attribute effects to specific interactions between microbes in the community.

## 4 Community-intrinsic properties

One of the most important aspects of studying microbial model communities is to capture and understand how microbes growing together interact in a way that is not predictable from how they grow in isolation. Indeed, if the constituent members of a community behave the same in the community setting as when grown alone, there is not really much point of studying them in a community context. Behaviors that are altered or specific to growth in community settings are often referred to as *community-intrinsic properties* [72] or, if they *only* appear when certain sets of microbes are growing together, *emergent community properties* [73].

### 4.1 Definitions

**Fig. 3:**
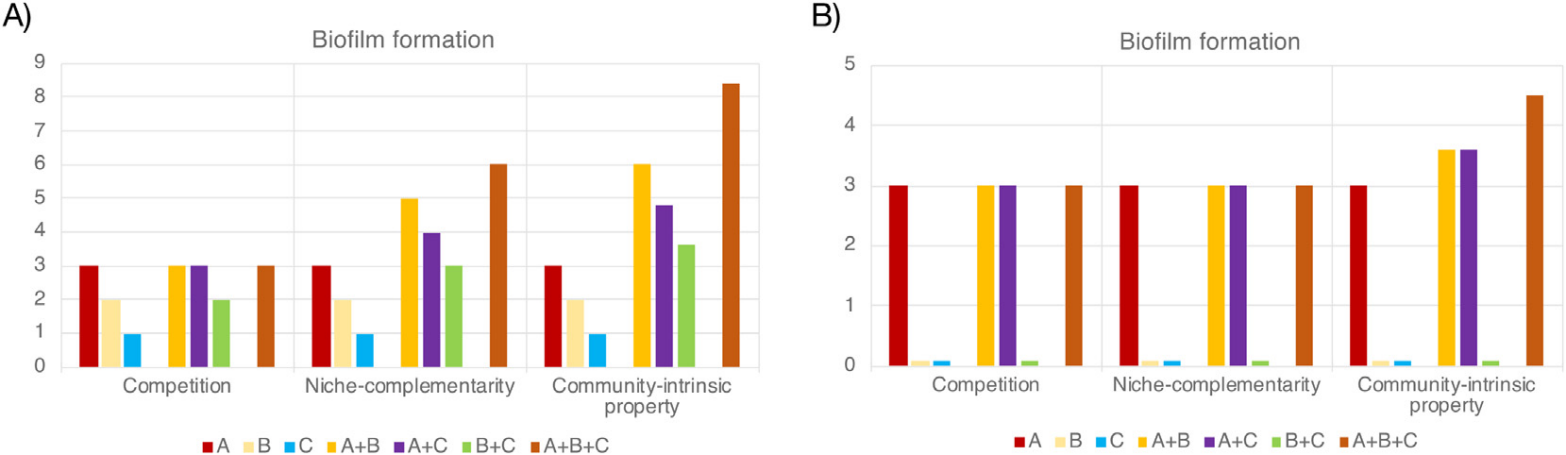
Example of different types of interactions among (fictional) biofilm-forming microbes. In (A), all three species are able to form biofilm on their own, albeit in different quantities, while in (B) only A is capable of forming biofilm on its own, while B and C are boosting the biofilm formation ability of A, akin to the situation in the THOR and SXMP model communities. In both (A) and (B), three scenarios are depicted. First, a scenario where all three species compete for the same resources is presented (i.e. the maximal biofilm formation is capped at 3). Second, a scenario where each species uses their own set of resources and therefore show limited interactions with the other species (niche complementarity) is shown. The final scenario is one of community-intrinsic behaviors, where interactions and cooperation among the three species result in more efficient resource utilization and increased biofilm output.

A community-intrinsic property could be defined as a behavior or outcome that cannot be predicted from the sum of the parts of the community [72]. Such a property could, for example, be stable oscillations in species abundances or predator–prey dynamics [74] but could also be an entity indicative of collective community function, such as increased biofilm formation in a community setting compared to what is produced by the individual species alone. The latter scenario will be used an example in the following text. Unfortunately, there are some additional complications to this definition. To begin, one could assume that the maximum possible biofilm formation (in the absence of any interaction) in a pair of two species would be the sum of the biofilm formation of the two species in the pair individually. However, in most cases this is an unreasonable assumption. Due to *competition* for resources, the total biofilm production would most likely be less than the sum of biofilm formation of the individual members, unless they are using completely different sets of nutrients and resources [24]. Such a scenario where the species do not share resources is referred to as *niche complementarity*, and happens when the species involved have non-overlapping requirements and therefore very minimal interactions despite occupying the same physical space [75], [76], [77]. This leads to the observation that if the biofilm formation when the pair is grown together is less than the sum of the production of each individual, it is likely that the two species have a competitive relationship (Fig. 3). At the same time, if the biofilm formation of the pair is roughly equal to sum of the individual productions, that indicates complementary of niches, which would mean no competition and no (substantial) interaction. This also leads to the interpretation that if biofilm formation in the pair is greater than the sum of the individual biofilm formation, there is an interaction effect (potentially, but not necessarily, mutualistic). This kind of greater-than-expected outcome will be used as the definition of a *community-intrinsic property* in this paper. Note that in many cases it may be that two species do have some type of synergistic interaction even in the competitive case, but it would be hard or maybe even impossible to separate that effect from the competition for resources that would also be taking place in parallel. Furthermore, as can be seen in Fig. 3B, it can sometimes be very hard to distinguish a competition scenario from a niche-complementary situation, particularly when communities get more complex.

The above reasoning can be extended to communities with more than two species. For example, in order to show a community-intrinsic property, a community with three members forming biofilm should have a total biofilm formation greater than the sum of all the biofilm produced by the constituents when grown alone. Should such a community also show biofilm production greater than the sum of all the pairs in order to be considered showing a community-intrinsic behavior? This is much less clear, especially as the community gets more complex with the addition of even more species. Here, it is useful to look at the average contribution of each species to the biofilm formation of the pair, across all pairs, rather than the total quantity of biofilm itself. If the increase of biofilm formation in the three-species community is greater than the sum of the amounts of biofilm for each pair divided by two (the average biofilm contribution per species in the pair), that would be an indicator of community-intrinsic properties (Fig. 3).

While this example deals with biofilm formation, the same reasoning can be extended to relative growth, production of metabolites or any measurable property of a community. It is important to note that while competition is a type of interaction, and almost by definition would be a community-intrinsic behavior, it is not a particularly interesting intrinsic property to study, as it is fairly self-evident that if you combine two species under resource limitation, they will compete for the available resources. Hence, competition will be considered separately from other community-intrinsic properties in this paper. That said, in communities with more than two species, differences between the expected degree of competition based on species-pairs grown together and the actual competition observed in the full community should be considered community-intrinsic properties, as they may be indicative of community protection from e.g. predation or chemical inhibition [52], [55].

### 4.2 How to quantify community-intrinsic properties?

Community-intrinsic properties of microbial communities are important in two different ways. First, their very existence hints at something fundamentally different about a cooperative lifestyle among microbes. Furthermore, the fact that some species seem to be better at inducing these community-intrinsic properties [52] shows that not all microbes are created equal in terms of cooperativity – some are more social than others. Second, obvious community-intrinsic properties can function as reporters for ongoing interactions in a community, both in the natural world and in the models we have discussed here. By measuring some easily observable community-intrinsic property (such as biofilm formation used in the example above), we can discern at what timepoint and under what conditions the members of a community show a high degree of interaction. Through assuming that strong interactions are likely to take place in the same timeframe (which may not always be true, but is more likely than the opposite), an easily measurable community-intrinsic property can be used to, for example, select optimal timepoints and conditions for an experiment.

Measuring these community-intrinsic properties can be tricky, especially in real time. Biofilm formation, for example, is fairly straightforward to measure. Typical assays for measuring biofilm formation include staining with crystal violet or other dyes, counting colony forming units from the biofilm on selective plates, coulter counting and flow cytometry [78]. However, these assays are quite intrusive and the results can take hours to obtain. This can be partially alleviated by carefully planning the experiment beforehand and using many parallel technical replicates of which some can be scarified for measurements of desired properties. Nevertheless, having some easily measured property, such as a change in pH, color of the media, or production of a fluorescent substance, makes on-the-fly measurements of the community interaction state much easier, and this is one of the many factors that should be weighted in when selecting a model community for studying a phenomenon (Table 1).

Another approach to measuring metabolic interactions in microbial communities is to label the metabolites themselves and follow their fate in the community. This can be achieved using stable-isotope tracing, in which a substrate such as glucose is labeled with ^13^C and then introduced as a nutrient source to the community [79], [80]. Due to metabolite exchanges, the ^13^C-labeled products that are produced in the community are indicative of which metabolic interactions that have taken place between the members. This allows for, e.g., flux analysis of these labeled compounds (^13^C-MFA), given that the members in the model community can be sorted or separated by strain and that the species have their metabolism well described [11]. However, the technique is at present somewhat complicated to scale up to model communities with many species due to these cell sorting requirements.

There is substantial literature on computational and statistical tools for modeling microbial community dynamics and fitting observations from microbiome data to these model predictions [11], [12], [13], and this paper will not address all the specific methods that can be used for these purposes. To some extent, any tool that has been devised to model or analyze complex microbiomes can be easily adapted to microbial model communities as well. There are, however, some community modelling approaches that lend themselves particularly well to model communities. For example, genome-scale metabolic models (GSMs) are much more easily extended from single-species assumptions to a community of well-known organisms with described genomes than to large and complex communities in natural settings. As such, GSMs provide a very useful computational framework for predicting model community behavior and detect deviations from expected interactions. Suitable computational approaches in this context have recently been reviewed by Frioux et al. [12] and Antoniewicz [11]. Furthermore, models based on ordinary differential equations (ODEs) are well equipped for modelling consumption and production of metabolites in model communities with few species or strains [13]. The ODE models capture the dynamics of co-cultured species using kinetic data. For example, an ODE model may be fed with information on how polysaccharides are converted to monosaccharides, how substrates are consumed and the individual growth rates, all of which can be estimated in monoculture setups. These predictions can then be experimentally tested in the model community setting. Finally, in the context of differentiating competition effects and community-intrinsic properties, it is useful to consider ecological models, including the generalized Lotka-Volterra model, often referred to as the predator–prey model, and models for synergisms, such as those proposed by Wintermute and Silver [20] and Mee et al. [60].

### 4.3 Examples of community-intrinsic properties in model communities

Due to the assorted definitions and unclear terminology around community properties, it is somewhat difficult to identify which microbial model communities that display clear and easily measurable community-intrinsic properties. In many microbial model communities, the only way of studying the community interactions is through the relative growth rates and yields of the community members (Table 1). Depending on the system, measuring relative growth can be a quite invasive procedure, which disrupts the community and/or takes time. Furthermore, relative growth is among the most sensitive properties to competition, as fundamental resources are generally shared and limited. Consequently, most directly growth-related interactions will be competitive, although exceptions exist (see below). As such, relative growth may not be an ideal endpoint for measuring community interactions.

A relatively large number of model communities use biofilm formation as a way to measure community interactions (SXMP, PPK, THOR, Pp-A [28], [45], [52], [53]). Of these, SXMP and THOR both clearly show community-intrinsic properties as defined in this paper, with one species dominating the biofilm in both cases and the other species boosting the biofilm production of that species (as in Fig. 3B). While both PPK and Pp-A also show increased biofilm formation in the community setting, it is less clear whether this is a community-intrinsic property or a case of niche-complementarity. Another type of growth-related readout is the ability to tolerate higher levels of antimicrobials, including antibacterial biocides. This property is shown by the PPK and PaSa-Wound [36] communities when grown together and can be useful for showing interactions in these model communities. However, this is also a readout that can be destructive and time-consuming to obtain. Similar ‘survival only in community settings’ types of properties can be used to detect community interactions in the Yeast-LAB (bacterial growth only in the presence of yeast), as well as in the auxotrophic mutant-based model communities [21], [48], [59], [60].

In a few of the model communities, production of some specific chemicals can be used as an indicator of community interactions. For example, in SF356 the degree of accumulation of oligosaccharides, acetate and ethanol is dependent on the presence of the community [38], in SaPa-CF *S. aureus* is only capable of fermentation when grown in the presence of *P. aeruginosa* [47], and in Cp-CBP ethanol production and cellulose fermentation is dependent on the presence of both the bacterium and a yeast species [39]. Such production of specific metabolites may be easier to measure than the relative growth of bacteria, and may also be more indicative of actual interaction between the community members. Finally, changes of e.g. pH and pigmentation may be possible to measure in real time, as in the Wolfe-Cheese community [46]. However, as can be seen here, it is generally not that easy to find community properties that can be measured non-destructively on-the-fly. Thus, methods such as fluorescent markers of the different members may help to obtain real time measurements of relative growth of the community members [21], [23], [28], [58], [81], [82].

## 5 Challenges and future work

### 5.1 Diversity of microbial model communities

One of the great challenges of research aiming to understand the complex interactions in natural systems is that these systems are most often highly diverse and therefore cover hundreds or thousands of microbial interactions. These interactions may look different in different systems and vary between combinations of microbial taxa. For the use of model communities to be of relevance in describing this myriad of interactions, there is a need for at least some degree of diversity of model communities, with models representing different types of environments and settings. At the same time, if specific models would be required to explain the community phenomena of every given setting, the ability to transfer any ecological knowledge from these model systems to full-scale microbial communities could be called into question. That said, if previous knowledge of biology provides any guidance, it seems likely that many of the findings regarding how interactions work in model communities would be translatable to a vast variety of full-scale ecological systems. For example, the ability to manipulate population dynamics using AI-2 quorum sensing was first discovered and explored in simple co-culture systems [83], [84], but was then transferred to mouse gut microbial communities [85], showing that there are indeed genetic mechanisms behind community interactions that are functional across scales. Nevertheless, it is likely that community interactions look different in planktonic settings compared to biofilms, in free-living communities compared to host-associated, in nutrient-rich compared to nutrient-deprived environments, and so on. This suggests that there is a need of some degree of diversity in terms of microbial model communities reflecting different microbial lifestyles.

Nevertheless, it is clear from this overview of available model systems that there is a considerable degree of overlap between systems, and that instead of focusing on a few well described systems, current model community efforts have been largely scattered across many different models. This is an inefficient use of scientific resources, and it would be useful if microbial model community researchers could come together and decide on a set of systems that would be considered ‘gold standard’ models. These should preferably be model systems we know much about already, that consist of microbes that are easy to grow and manipulate, that have relevance in real-world applications, that show community properties that are indicative of healthy microbiome function, and that are highly reproducible between labs (see also [40]). The model communities should also be easy to adapt to different scales and allow high-throughput experiments. There are only a limited number of model communities described in this paper that live up to all these criteria, but good candidates would be the PPK [28], Yeast-LAB [48], SXMP [45], and THOR [52] model communities, which contain easily grown species, have already been evaluated for at least some more complex community behaviors, have simple readouts and at least some degree of relevance for different natural systems (biofilms, fermented food and soil, respectively). For human-associated communities, the two model communities comprising the *P. aeruginosa* and *S. aureus* species pair – PaSa-Wound and SaPa-CF – represent the best-studied models [36], [47]. However, both these models represent a largely disruptive pathogenic situation. The two gut microbial model communities (Venturelli-Gut and Gutierrez-Gut) represent more normal conditions; however, they both are fairly complex, have less crystal-clear readouts and comprise several species that are somewhat problematic to grow under laboratory conditions [22], [32]. It should be determined if such complex models are necessary to accurately describe the most important interactions in the human gut and what could potentially be learned from simpler model communities.

### 5.2 Controlled settings are crucial

Another challenge related to model communities is that many community-intrinsic properties may be highly dependent on specific environmental conditions. For example, community-intrinsic properties may only appear in certain media [37], [46], [52], or may require that the environment is deprived on some key element or compound [51], [53], [59]. It is also common that the communities require specific substrates for e.g. biofilm formation [36], [58]. In most cases, such specifics are less important in single-species culture than in multi-species communities. When a strain is grown alone, it can use up all resources and does not have to compete for space and nutrients. This means that differences in growth temperature or substrate may have effects on growth that are barely discernable, as the final yield is the same regardless of these minor changes. However, when a strain has to compete for resources with other species and strains, constraints such as optimal growth temperature, osmotic pressure, pH, available substrates, as well as initial inoculum size, start having large downstream effects on growth relative to the other strains in the community. Even small changes in growth temperature have been shown to dramatically alter biofilm formation ability in the THOR community, for example (Burman et al., unpublished data). It is therefore instrumental to i) agree upon standards for growing model communities in the lab (although these standards can be different for different purposes), ii) state exactly what inoculum sizes and growth conditions were used in community experiments (not only using “room temperature”, for example), iii) relate community properties to the growth of the individual members of the community, and iv) carefully study how different model communities grow under different conditions and how temperature, media, starting inoculum, substrates etc. affect the expression of community properties and community dynamics. In order for such experiments to be worthwhile, it would be important to unite around a smaller number of priority model communities – the ‘gold standard’ communities discussed above.

### 5.3 Common definitions, terms and frameworks

Similar to the need for agreement on growth conditions for microbial model communities, there is a need to settle on a common set of definitions for phenomena in model communities. It would, for example, be useful to have agreed-upon definitions of community-intrinsic and emergent properties, niche complementarity and competitive interactions. As pointed out by Madsen et al. [72], it is also important to determine what constitutes a reasonable null model for how a non-interacting community would behave that encompasses more complex scenarios than those shown in Fig. 3. Such a null model could easily get complicated, particularly as the number of community members grow. Therefore, the development of such models is an entire area of research in itself, where experimentalists, computational biologists and ecologists need to work together to achieve an agreement on a common framework for community interactions in model systems, in order to allow the field to progress as efficiently as possible. The use of null models is common in addressing community assembly [86], [87], but is not as well established for microbial interaction networks [11]. Only when a framework for generating relevant null models is in place will it be possible to fully take advantage of the power of computational approaches to predict the behavior of microbial communities [88].

### 5.4 Disentangling complexity requires an understanding of simplicity

The endeavor to tease apart interactions in complex microbial communities needs to start small. Microbial model communities of different scales have contributed to our understanding of microbial predator–prey relationships [23], [30], [55], resource partitioning [35], [48], [53], [57], pathogenicity [37], [47], antibiotic tolerance [28], [89], [90], resistance to stress [28], [31], as well as the conditions that trigger the production of secondary metabolites [26], [46], [52]. This, however, is just scratching the surface of what model communities could reveal about community dynamics; it is apparent that there is a plethora of biological phenomena related to interactions between microbes to explore, and model communities provide a means to start assessing these interactions and the mechanisms behind them. The model systems offer an opportunity to track down the genetic mechanisms behind interactions and predict how these mechanisms would play out in more complex systems. For example, by studying disturbances to model communities and the genes that are important for resilience to disruption, we can predict how complex natural communities in various settings would respond to the same perturbations. Deviation from these predictions can then be interpreted in a functional context, for example by investigating if there is functional redundancy in the more diverse and complex natural community [91]. That question can really only be tackled knowing the genetic mechanisms behind, in this case, community stability. Knowledge of how to maintain community interactions could be instrumental in treating diseases with diffuse causes connected to the human microbiome, such as IBS and IBD [92], [93], [94], [95]. Despite all this, one of the major shortcomings of microbial model communities is that there have been very few attempts to translate findings from model communities into real-world complex communities. There is a clear need to verify the interactions claimed to be important for microbial communities and their mechanisms in complex systems in order to ascertain the relevance of the microbial model community systems we already use, if not else to determine the limitations of this approach to yield generalizable conclusions. Furthermore, by understanding which genes are responsible for interactions in biotechnologically important communities, we could potentially either simplify these consortia using genetic engineering, or increase their total productivity by adding beneficial community members [96]. Identifying these additional members that would allow for more efficient resource utilization (from a human perspective) requires a well-understood model community to pinpoint relevant interactions. Common to these endeavors is the need to understand genetic and molecular mechanisms in less complex settings before proceeding to disentangle the many intricate relationships in full-scale microbial communities in natural environments.

## 6 Summary and outlook

As stated from the start, natural microbial ecosystems are complex. Our best shot at understanding this complexity is to reduce it into components we can more easily disentangle and then figure out the larger picture piece by piece, sometimes extrapolating from the limited data we have. In order to do so, microbial model communities are essential instruments to learn about metabolic interactions, genetic mechanisms and ecological principles governing and structuring communities. There already exists a range of microbial model communities of varying complexity, designed to answer different questions and reflect different phenomena, environments and applications. Choosing the right model community for the questions asked is key to achieve relevant results, but is no easy task. A few suggestions of model communities suitable for different types of research questions are given in Table 2. This review can function as a guide in this selection process, but does by no means provide a universal answer to all situations where model communities would be useful. That said, focusing on well-studied models and centralizing around a smaller number of experimental systems would be highly useful. Some of the best studied models for microbial communities are PPK [28], Yeast-LAB [48], SXMP [45], and THOR [52]. All of these community models also consist of species easily grown in the lab, have been evaluated for community behaviors, have simple readouts and are of relevance for natural ecosystems. There are fewer really good and well-studied models for human-associated communities, so unfortunately the choice of model in that context is much more open at this time. Finally, there is a need for agreement and standardization of growth conditions for microbial model communities, definitions of community-specific phenomena, and frameworks for how (non–) interacting communities would behave. All this would require scientists across disciplines to come together in an effort to reduce the complexity of the universe of model systems itself.

**Table 2:** Suggested model communities for different types of research questions.

Research question	Suggested model communities	References
(1) Sharing of metabolites and partitioning of resources	SeMeCo, Ec-Coculture, Ec-Crossfeeding	[21], [59], [60]
(2) Competitive vs. cooperative interactions in microbial communities	C-S-R, Ec-Predator, Venturelli-Gut, Gutierrez-Gut	[22], [23], [32], [55]
(3) Identifying genes governing microbial interactions	THOR, SXMP, Kim-Soil; see also the approach of Wintermute and Silver, and the models under question (10)	[20], [45], [51], [52]
(4) How interactions are affected by environmental changes	PPK, THOR, SXMP, PaSa-Wound, Kim-Soil	[28], [36], [45], [51], [52]
(5) The links between taxonomic and functional diversity	The approach of Goldford et al.	[69]
(6) The effect of stressors (including invasion by non-native species) on community stability	PPK, THOR, SXMP, PaSa-Wound, Kim-Soil, Venturelli-Gut, Gutierrez-Gut	[22], [28], [32], [36], [45], [51], [52]
(7) The importance of keystone species for functional stability	Wolfe-Cheese, Venturelli-Gut, Gutierrez-Gut	[22], [32], [46]
(8) The evolution of mutualism in microbial communities	Harcombe-ESM, ZmEc-Mutualism	[35], [57]
(9) Identification of factors that allow pathogens to outcompete commensal bacteria	PPK, Venturelli-Gut, Gutierrez-Gut, CWPB	[22], [28], [32], [37]
(10) Improving yields of certain desired compounds	Yeast-LAB, SF356, Pp-A, Cp-CBP, Clostridium-Syntrophy, Wolfe-Cheese	[38], [39], [46], [48], [50], [53]

## CRediT authorship contribution statement

**Johan Bengtsson-Palme:** Conceptualization, Methodology, Investigation, Funding acquisition.

## Declaration of Competing Interest

The author declares that he has no known competing financial interests or personal relationships that could have appeared to influence the work reported in this paper.
